# Assessing the protective role of allergic disease in gastrointestinal tract cancers using Mendelian randomization analysis

**DOI:** 10.1111/all.14616

**Published:** 2020-10-21

**Authors:** Shuai Yuan, Mathew Vithayathil, Siddhartha Kar, Paul Carter, Amy M. Mason, Shao‐Hua Xie, Stephen Burgess, Susanna C. Larsson

**Affiliations:** ^1^ Unit of Cardiovascular and Nutritional Epidemiology Institute of Environmental Medicine Karolinska Institutet Stockholm Sweden; ^2^ MRC Cancer Unit University of Cambridge Cambridge UK; ^3^ MRC Integrative Epidemiology Unit Bristol Medical School University of Bristol Bristol UK; ^4^ Department of Public Health and Primary Care University of Cambridge Cambridge UK; ^5^ British Heart Foundation Cardiovascular Epidemiology Unit Department of Public Health and Primary Care University of Cambridge Cambridge UK; ^6^ National Institute for Health Research Cambridge Biomedical Research Centre University of Cambridge and Cambridge University Hospitals Cambridge UK; ^7^ Upper Gastrointestinal Surgery Department of Molecular Medicine and Surgery Karolinska Institutet Karolinska University Hospital Stockholm Sweden; ^8^ MRC Biostatistics Unit University of Cambridge Cambridge UK; ^9^ Department of Surgical Sciences Uppsala University Uppsala Sweden

To the Editor,

Immune hypersensitivity featured among allergic individuals has been proposed to enhance immune surveillance, thereby inhibiting abnormal cell growth and thus reduce cancer risk.^1^, ^2^ Similarly, the prophylaxis theory suggests that allergy symptoms may prevent cancer development by removing potential carcinogens.^2^ This hypothesis envisages a preventive role specifically for cancers of tissues that interface with the external environment, such as gastrointestinal tract cancers.^2^ A reduced risk of gastrointestinal tract cancer among individuals with self‐reported allergic conditions has been observed in several but not all observational studies.^3^, ^4^ However, whether the associations are causal remains unclear as observational studies are prone to residual confounding. Therefore, we conducted a Mendelian randomization study^5^ to determine the causal role of genetic liability to allergic disease in esophageal, gastric, and colorectal cancers.

One hundred and thirty‐six single‐nucleotide polymorphisms (SNPs) associated with at least one allergic disease (asthma, allergic rhinitis, or eczema) at *P* < 3×10^−8^ in 180 129 cases and 180 709 non‐cases of European ancestry were considered as instrumental variables.^6^ The 136 SNPs were independent and not in linkage disequilibrium defined by distance >1 Mb and *r* ^2^ < 0.02. The SNPs explained around 2.6% of phenotypic variance. Summary‐level data for the associations of those SNPs with esophageal, gastric, and colorectal cancers were obtained from the UK Biobank, BioBank Japan, and FinnGen consortium. The main analysis was based on the multiplicative random‐effects inverse‐variance weighted method, and estimates from the different studies were combined using fixed effects meta‐analysis. The inverse‐variance weighted method under a fixed effects model and the weighted median, MR‐Egger regression, and MR‐PRESSO methods were employed as sensitivity analyses. The *I* ^2^ (%) statistic and Cochrane's *Q* value were calculated to assess heterogeneity among estimates of individual SNPs. We considered associations with *P* values below .017 (where *P* = .05/3) to represent strong evidence of causal associations and associations with *P* values below .05 but above .017 as suggestive evidence of associations.

Genetic liability to allergic disease was associated with lower risk of the gastrointestinal tract cancers in the meta‐analysis of UK Biobank, BioBank Japan, and FinnGen, though the association with gastric cancer was not statistically significant after correcting for multiple testing (Figure 1). The combined odds ratio was 0.85 (95% confidence interval, 0.75, 0.97; *P* = .016) for esophageal cancer, 0.90 (95% confidence interval, 0.82, 0.98; *P* = .020) for gastric cancer, and 0.91 (95% confidence interval, 0.86, 0.96; *P* = .001) for colorectal cancer for one unit increase in log‐transformed odds of allergic disease. The associations were consistent across data sources with the exception of the estimate for gastric cancer in FinnGen which was above one but with a broad confidence interval that overlapped the estimates in UK Biobank and BioBank Japan. Results were consistent in sensitivity analyses for all three cancer outcomes (Table 1).

**Figure 1:**
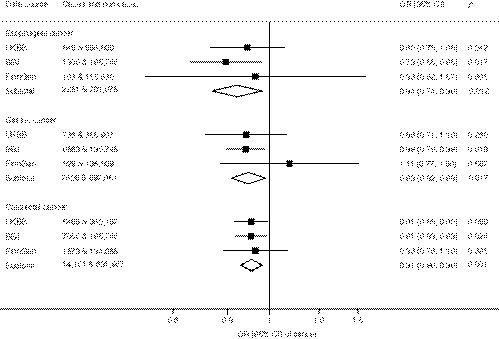
Associations of genetic predisposition to allergic disease with esophageal, gastric, and colorectal cancer. BBJ, indicates BioBank Japan; CI, confidence interval; OR, odds ratio; UKBB, UK Biobank

**Table 1:** Associations of genetic predisposition to allergic disease with esophageal, gastric, and colorectal cancer in sensitivity analyses

	Esophageal cancer			Gastric cancer			Colorectal cancer		
	OR	95% CI	*P*	OR	95% CI	*P*	OR	95% CI	*P*
UK Biobank									
IVW‐fixed effects method	0.89	0.73, 1.08	.242	0.88	0.72, 1.09	.253	0.91	0.84, 0.98	.015
Weighted median method	0.92	0.69, 1.23	.571	0.84	0.61, 1.15	.270	0.96	0.84, 1.08	.489
MR‐Egger regression	1.01	0.6, 1.68	.976	1.23	0.71, 2.13	.468	0.91	0.73, 1.15	.442
MR‐PRESSO method^a^	n/a	n/a	n/a	n/a	n/a	n/a	0.90	0.83, 0.98	.012
Heterogeneity^b^	*I*^2^ = 0%; Cochrane's *Q* = 114; *P*_het_ = .877			*I*^2^ = 0%; Cochrane's *Q* = 137; *P*_het_ = .393			*I*^2^ = 21%; Cochrane's *Q* = 168; *P*_het_ = .021		
Pleiotropy^c^	Intercept = −0.007; *P*_pleiotropy_ = .601			Intercept = −0.018; *P*_pleiotropy_ = .208			Intercept = 0.000; *P*_pleiotropy_ = .944		
BioBank Japan									
IVW‐fixed effects method	0.79	0.66, 0.96	.017	0.88	0.81, 0.96	.004	0.91	0.83, 0.99	.022
Weighted median method	0.65	0.49, 0.86	.003	0.94	0.82, 1.07	.332	0.87	0.76, 0.99	.034
MR‐Egger regression	0.65	0.37, 1.14	.130	0.83	0.61, 1.13	.237	0.84	0.66, 1.08	.181
MR‐PRESSO method^a^	n/a	n/a	n/a	0.93	0.84, 1.02	.138	n/a	n/a	n/a
Heterogeneity^b^	*I*^2^ = 0%; Cochrane's *Q* = 92; *P*_het_ = .833			*I*^2^ = 31%; Cochrane's *Q* = 154; *P*_het_ = .002			*I*^2^ = 3%; Cochrane's *Q* = 109; *P*_het_ = .407		
Pleiotropy^c^	Intercept 0.011; *P*_pleiotropy_ = .453			Intercept = 0.003; *P*_pleiotropy_ = .682			Intercept = 0.004; *P*_pleiotropy_ = .541		
FinnGen consortium									
IVW‐fixed effects method	0.93	0.52, 1.67	.803	1.11	0.79, 1.56	.537	0.93	0.79, 1.09	.352
Weighted median method	0.79	0.34, 1.85	.586	1.21	0.72, 2.02	.477	1.11	0.88, 1.4	.38
MR‐Egger regression	0.30	0.06, 1.43	.129	1.00	0.37, 2.67	.999	0.88	0.55, 1.39	.578
MR‐PRESSO method^a^	n/a	n/a	n/a	n/a	n/a	n/a	n/a	n/a	n/a
Heterogeneity^b^	*I*^2^ = 0%; Cochrane's *Q* = 81; *P*_het_ = .998			*I*^2^ 14%; Cochrane's Q 140; *P*_het_ = .103			*I*^2^ 16%; Cochrane's Q 143; *P*_het_ = .078		
Pleiotropy^c^	Intercept = 0.061; *P*_pleiotropy_ = .127			Intercept 0.006; *P*_pleiotropy_ = .818			Intercept 0.003; *P*_pleiotropy_ = .797		

^a^ There was no corrected estimate (n/a) if no outlier was identified. One outlier was identified and removed in the analysis of colorectal cancer based on UK Biobank (*P* value for the distortion test was.815) and two outliers were identified and removed in the analysis of gastric cancer based on Biobank Japan (*P* value for the distortion test was.137).

^b^ We observed significant heterogeneity among used SNPs in the analysis of colorectal cancer based on UK Biobank and gastric cancer based on Biobank Japan (*P*_het_ < .05).

^c^ Pleiotropy was measured by the intercept from MR‐Egger regression. Pleiotropy was not detected in any analysis (all *P > *.05).

A potential limitation arises from the fact that we used an instrument for liability to allergic disease that was developed using genetic data from individuals of European ancestry and applied to a Japanese population. However, a consistent effect of risk alleles of SNPs for allergic disease on asthma in BioBank Japan indicates a high validity of used instrumental variables and a negligible population bias. In addition, a robust pattern of established associations in both Asian and European populations suggested that the associations were portable across populations of distinct ancestries. A further limitation of this work is that we cannot make specific inferences about the impact of food allergies on cancer risk.

In summary, this MR study supports a protective effect of allergic disease against esophageal and colorectal cancer and possibly gastric cancer. This finding may be useful for further laboratory studies to understand the immunological pathways involved in gastrointestinal tract carcinogenesis.

## CONFLICT OF INTEREST

Dr. Mason reports grants from the EC‐Innovative Medicines Initiative (BigData@Heart) and the National Institute for Health Research (Cambridge Biomedical Research Centre at the Cambridge University Hospitals NHS Foundation Trust).

## ETHICAL APPROVAL

All studies included in cited genome‐wide association studies had approved by a relevant review board. The present MR analyses were approved by the Swedish Ethical Review Authority (2019‐02793).

## Supporting information

Supplementary Material
