# Supplementary material for: Assessing the protective role of allergic disease in gastrointestinal tract cancers using Mendelian randomization analysis
Source: Allergy. 2020 Oct 21;76(5):1559–62. doi: 10.1111/all.14616 (PMC8411419; doi:10.1111/all.14616)
Supplement: Supplementary file 1 — Supplementary Material [file ALL-76-1559-s001.docx]

**Study hypothesis**

A reduced risk of gastrointestinal tract cancer among individuals with self-reported allergic conditions, such as asthma, allergic rhinitis, and eczema, has been found in several but not all observational studies.^1,2^ Nevertheless, due to potential methodological limitations, such as residual confounding and reverse causality, embedded in observational studies, it remains inconclusive whether allergic disease exerts a causal effect on gastrointestinal tract cancer. For example, infection of helicobacter pylori, alcohol consumption, obesity and socioeconomic status showed associations with both allergy ^3^ and certain types of gastrointestinal tract cancers.^4,5^ Given that few studies took all possible confounders into consideration, the observed inverse association between allergic disease and gastrointestinal tract cancer could be skewed by the unobserved confounding. A clear appraisal of the causal link from allergic disease to major gastrointestinal cancers is of great importance not only for building accurate risk prediction models to potentially discriminate high-risk individuals from the general population, but also shedding light on the etiology of these cancers, thereby facilitating prevention and treatment strategies.

**A brief introduction to Mendelian randomization**

Through the use of genetic variants as instruments for an exposure (e.g. genetic liability to allergic disease), the Mendelian randomization (MR) analysis can strengthen the causal inference for an exposure-outcome association by minimizing residual confounding and bias due to reverse causality.^6,7^ The rationale for minimization of residual confounding is that genetic variants are randomly allocated at conception, and therefore, genetically proxied exposures are generally not correlated with other confounding traits. The MR design can also minimize potential bias due to reverse causality because alleles are fixed at birth and cannot be modified by the onset or progression of the disease.^6,7^ Therefore, if a genetic variant robustly associated with an exposure is also associated with an outcome and the effect on the outcome is not stronger than that on the exposure, this offers evidence that the exposure exerts causal effects on the outcome but not *vice versa*. While allergic disease is in itself a disorder, genetic liability to allergic disease can be considered an exposure in the MR setting.

**Detailed information on genome-wide association studies on allergic disease**

One hundred and thirty-six single-nucleotide polymorphisms associated with at least one allergic disease (asthma, allergic rhinitis or eczema) at *p* <3×10^-8^ in a meta-analysis of 13 genome-wide association studies including 180,129 cases (83,827 cases with one allergic disease, 30,418 cases with two allergic diseases, 6418 cases with three allergic diseases and 59,466 unclassifiable cases) and 180,709 non-cases of European ancestry were considered as instrumental variables.^8^ The analyses had been adjusted for age, sex and population principal components. The mean age of participants in the meta-analysis was around 52 years and there were 52% women. The 136 SNPs were independent and not in linkage disequilibrium defined by distance >1Mb and *r^2^* <0.02. All SNPs together explained around 2.6% of phenotypic variance. To evaluate the applicability of the instrumental variables for allergic disease identified in individuals of European descent for the Japanese population, we examined the effect of the genetic instrument on risk of asthma using data from BioBank Japan. A significant elevated risk of asthma (OR 2.38; 95% CI, 2.06, 2.74; *p*=1.1×10^-32^) among Japanese individuals with risk alleles of allergic disease indicated the validity of the genetic instrument.

**Detailed information on data sources of cancers**

**UK Biobank**

The UK Biobank study recruited around 500,000 adults aged 40 to 69 years in 2006-2010.^9^ In the present analysis, we included 367,643 individuals of European ancestry after excluding related individuals (third-degree relatives or closer), low call rate, and excess heterozygosity (3 or more standard deviations from the mean). Participants were followed up until 31 March 2017 or the date of death (recorded until 14 February 2018) in the present study with a median follow-up of around 8.0 years. Cancer outcomes were defined based on registry data and self-reported information validated by nurse interview. We calculated genetic association estimates for cancer (beta coefficients and corresponding standard errors) using logistic regression with adjustment for age, sex and the first ten genetic principal components.

**BioBank Japan**

We obtained summary-level estimates from BioBank Japan and the FinnGen consortium. BioBank Japan is a genetic study with a total of 212,453 Japanese individuals.^10^ Individuals with a sample call rate of <0.98 and outliers from East Asian clusters and genetic variants with call rate < 99%, *p* value for Hardy–Weinberg equilibrium <1.0×10^-6^, and fewer than five heterozygotes were excluded in the quality control stage of genome-wide association analyses. All samples in 1KG Phase3 (version 5) were used as the reference population in imputation stage and analyses were adjusted for age, sex and the first five genetic principal components.

**The FinnGen consortium**

The FinnGen consortium encompasses 135,638 Finnish individuals in the last publicly available (R3) data release. The consortium excluded participants with ambiguous gender, high genotype missingness (>5%), excess heterozygosity (±4 standard deviation) and non-Finnish ancestry and genetic variants with high missingness (>2%), low Hardy-Weinberg equilibrium *p*-value (*p*<5×10^-6^) and minor allele count <3. The population specific SISu v3 reference panel was used in the imputation stage and genome-wide association tests adjusted for age, sex, the first ten genetic principal components, and genotyping batch. Detailed information about FinnGen is available on its website (<https://www.finngen.fi/>).

**Detailed description of statistical analysis**

The main analysis was based on the multiplicative random-effects inverse-variance weighted method,^11^ and estimates from the different studies were combined using fixed-effects meta-analysis. The inverse-variance weighted method under a fixed-effects model and the weighted median,^12^ MR-Egger regression^13^ and MR-PRESSO^14^ methods were employed as sensitivity analyses to examine the robustness of results and correct for potential pleiotropy. The F-statistic was estimated to measure the strength of the genetic instrument. The *I^2^* (%) statistic^15^ and Cochrane’s Q value was calculated to assess heterogeneity among estimates of individual SNPs. Odds ratios (ORs) and corresponding confidence intervals (CIs) of cancer were scaled to one-unit increase in log-transformed odds of allergic disease. To account for multiple testing, we considered associations with *p* values below 0.017 (where *p* = 0.05/3 (3 cancer outcomes)) to represent strong evidence of causal associations, and associations with *p* values below 0.05 but above 0.017 as suggestive evidence of associations. All analyses were two-sided and performed using the mrrobust package^16^ in Stata/SE 15.0 (Stata Statistical Software: Release 15. College Station, TX: StataCorp LLC.), and the MRPRESSO^14^ and TwoSampleMR^17^ packages in R Software 3.6.0 (R Core Team. R Foundation for Statistical Computing. Vienna, Austria. 2019. https://www.R-project.org).

**Supplementary results**

The number of SNPs available as genetic instruments for allergic condition in the UK Biobank, BioBank Japan and FinnGen datasets was 134, 110 and 121, respectively. The F-statistic for individual SNPs was over 10 (ranging from 32 to 296) and the average F-statistic was around 71. Results of sensitivity analyses are shown in Table 1. Overall, results were consistent in sensitivity analyses for all three cancer outcomes. We observed significant heterogeneity among estimates across SNPs in the analysis of colorectal cancer based on UK Biobank and gastric cancer based on BioBank Japan (*p*_het_<0.05). However, no pleiotropy was detected by MR-Egger regression (all *p* for intercept > 0.05). One outlier SNP was identified in the analysis of colorectal cancer based on UK Biobank and two outlier SNPs were identified in the analysis of gastric cancer based on BioBank Japan. Outlier-corrected associations persisted and the *p* values for two distortion tests were over 0.05, indicating no significant difference between estimates derived from analysis before and after removal of outlier SNPs.

**Discussion on findings**

The present MR study found support for causal inverse associations between genetic liability to allergic disease and risk of esophageal and colorectal cancers using data from three independent populations. Genetic liability to allergic disease also showed a suggestive inverse association with risk of gastric cancer. Our findings are overall consistent with results of most traditional observational studies. A systematic review study that comprehensively summarized the association between allergies and site-specific cancers found that individuals with any type of allergy had a lower risk of esophageal, gastric and colorectal cancer.^18^ The associations were subsequently replicated in studies with a large sample size,^1^ especially for colorectal cancer.^2,19,20^ In a meta-analysis of 12 studies including 515,379 individuals and 10,345 colorectal cancer cases, the relative risk for the association between allergic disease and colorectal cancer was 0.88 (95% CI 0.83, 0.92) ^2^. However, with regard to esophageal and gastric cancer, a hospital-based cohort study with 92,986 asthma patients found a modest increased risk of these cancers among asthma patients compared with the general population, but the excessive risks were caused by a higher prevalence of gastro-esophageal reflux among asthma patients.^21^ The discrepancy in results for esophageal cancer might be caused by different effects of allergic disease on esophageal squamous cell carcinoma and esophageal adenocarcinoma. The protective effect of allergy may be stronger for or restricted to esophageal squamous cell carcinoma^1^ The stronger association observed between allergic disease and esophageal cancer in BioBank Japan than in the UK Biobank and FinnGen consortium in the present study may also support this hypothesis considering the higher prevalence of esophageal squamous cell carcinoma in the Japanese population than in European populations.^22^

Two major hypotheses have been proposed to explain the link between allergic disease and reduced risk of gastrointestinal tract cancers. The mainstay one is the immunosurveillance hypothesis assuming that individuals with allergic disease own a generally enhanced immune responsiveness, thereby having the propensity to detect and eradicate dysregulated cells. In detail, atopic allergens can enter human body via several systems at numerous points, including the respiratory system and the digestive system, where mast cells, basophils and eosinophils are omnipresent ^23^. Type I immunoglobulin E-mediated hypersensitivity reactions will be activated by this process and so does an excessive T-helper cell type II response due to the overstimulation of eosinophils and mast cells. Enhanced permeability and inflammation after mast cells degranulating, immunoglobulin E antibody-dependent cellular cytotoxicity in precancerous and cancerous cells and direct anticancer effects may help clear dysregulated cells and limit the growth of abnormal cells ^24,25^. In addition, an increased level of tumor associated tissue eosinophils has been showed associations with lower rates of venous invasion, lymph node metastases and better clinic outcome in esophageal squamous cell carcinoma ^26^. The other hypothesis is the prophylaxis hypothesis, which suggests that the physical effect of allergic responses and inflammation in mucosal surfaces removes mutagenic triggers, such as sneezing or coughing out the microbial carcinogen Aflatoxin B1 produced by the fungus Aspergillus flavus before the start of malignant transformation ^24^.

There are several strengths and limitations of the present study. The major merit is the MR design which strengthens the causal inference in the associations of allergic disease with three major gastrointestinal tract cancers. The associations for esophageal and colorectal cancer were coherent in three independent population, thereby providing strong support for that the findings for these site-specific cancers are causal. Moreover, a high consistency across MR analyses indicated a negligible distortion by potential pleiotropy. However, the finding for gastric cancer needs verification considering the somewhat inconclusive results across studies. A part of asthma cases was not allergic but caused by other factors, which might have affected our findings. However, only around 5.6% of cases were diagnosed with only asthma.^8^ This means that the vast majority of recruited cases had allergic conditions and the estimates of used SNPs could appropriately represent the genetic effects of used instruments on allergic conditions. The influence of allergic conditions on cancer risk may differ for allergic rhinitis, asthma, and eczema ^1^. Whether the observed associations in this study were driven by a certain allergic disease needs to be investigated. Nevertheless, given a high comorbidity of different allergic diseases ^8^, an overall causal effect revealed by our finding is of great significance in the public health and clinical setting. A further limitation of this work is that we cannot make specific inferences about the impact of food allergies on cancer risk. Whether food allergy has protective effect as allergic diseases on these cancers is of great interest and deserves examination in future studies.

**References**

1. D'Arcy M, Rivera DR, Grothen A, Engels EA. Allergies and the Subsequent Risk of Cancer among Elderly Adults in the United States. *Cancer Epidemiol Biomarkers Prev.* 2019;28(4):741-750.

2. Ma W, Yang J, Li P, Lu X, Cai J. Association between allergic conditions and colorectal cancer risk/mortality: a meta-analysis of prospective studies. *Sci Rep.* 2017;7(1):5589.

3. Chen Y, Blaser MJ. Inverse associations of Helicobacter pylori with asthma and allergy. *Arch Intern Med.* 2007;167(8):821-827.

4. Peek RM, Jr., Blaser MJ. Helicobacter pylori and gastrointestinal tract adenocarcinomas. *Nat Rev Cancer.* 2002;2(1):28-37.

5. Cui Y, Hill AW. Atopy and Specific Cancer Sites: a Review of Epidemiological Studies. *Clin Rev Allergy Immunol.* 2016;51(3):338-352.

6. Burgess S, Thompson SG. *Mendelian randomization: methods for using genetic variants in causal estimation.* CRC Press; 2015.

7. Smith GD, Ebrahim S. 'Mendelian randomization': can genetic epidemiology contribute to understanding environmental determinants of disease? *Int J Epidemiol.* 2003;32(1):1-22.

8. Ferreira MA, Vonk JM, Baurecht H, et al. Shared genetic origin of asthma, hay fever and eczema elucidates allergic disease biology. *Nat Genet.* 2017;49(12):1752-1757.

9. Sudlow C, Gallacher J, Allen N, et al. UK biobank: an open access resource for identifying the causes of a wide range of complex diseases of middle and old age. *PLoS Med.* 2015;12(3):e1001779.

10. Ishigaki K, Akiyama M, Kanai M, et al. Large-scale genome-wide association study in a Japanese population identifies novel susceptibility loci across different diseases. *Nat Genet.* 2020;52(7):669-679.

11. Burgess S, Bowden J, Fall T, Ingelsson E, Thompson SG. Sensitivity Analyses for Robust Causal Inference from Mendelian Randomization Analyses with Multiple Genetic Variants. *Epidemiology.* 2017;28(1):30-42.

12. Bowden J, Davey Smith G, Haycock PC, Burgess S. Consistent Estimation in Mendelian Randomization with Some Invalid Instruments Using a Weighted Median Estimator. *Genet Epidemiol.* 2016;40(4):304-314.

13. Bowden J, Davey Smith G, Burgess S. Mendelian randomization with invalid instruments: effect estimation and bias detection through Egger regression. *Int J Epidemiol.* 2015;44(2):512-525.

14. Verbanck M, Chen CY, Neale B, Do R. Detection of widespread horizontal pleiotropy in causal relationships inferred from Mendelian randomization between complex traits and diseases. *Nat Genet.* 2018;50(5):693-698.

15. Higgins JP, Thompson SG. Quantifying heterogeneity in a meta-analysis. *Stat Med.* 2002;21(11):1539-1558.

16. Spiller W, Davies NM, Palmer TM. Software application profile: mrrobust—a tool for performing two-sample summary Mendelian randomization analyses. *International Journal of Epidemiology.* 2019;48(3):6.

17. Hemani G, Zheng J, Elsworth B, et al. The MR-Base platform supports systematic causal inference across the human phenome. *Elife.* 2018;7.

18. Merrill RM, Isakson RT, Beck RE. The association between allergies and cancer: what is currently known? *Ann Allergy Asthma Immunol.* 2007;99(2):102-116; quiz 117-109, 150.

19. Prizment AE, Folsom AR, Cerhan JR, Flood A, Ross JA, Anderson KE. History of allergy and reduced incidence of colorectal cancer, Iowa Women's Health Study. *Cancer Epidemiol Biomarkers Prev.* 2007;16(11):2357-2362.

20. Tambe NA, Wilkens LR, Wan P, et al. Atopic allergic conditions and colorectal cancer risk in the Multiethnic Cohort Study. *Am J Epidemiol.* 2015;181(11):889-897.

21. Ye W, Chow WH, Lagergren J, et al. Risk of adenocarcinomas of the oesophagus and gastric cardia in patients hospitalized for asthma. *Br J Cancer.* 2001;85(9):1317-1321.

22. Wong MCS, Hamilton W, Whiteman DC, et al. Global Incidence and mortality of oesophageal cancer and their correlation with socioeconomic indicators temporal patterns and trends in 41 countries. *Sci Rep.* 2018;8(1):4522.

23. Rigoni A, Colombo MP, Pucillo C. Mast cells, basophils and eosinophils: From allergy to cancer. *Semin Immunol.* 2018;35:29-34.

24. Josephs DH, Spicer JF, Corrigan CJ, Gould HJ, Karagiannis SN. Epidemiological associations of allergy, IgE and cancer. *Clin Exp Allergy.* 2013;43(10):1110-1123.

25. Jensen-Jarolim E, Achatz G, Turner MC, et al. AllergoOncology: the role of IgE-mediated allergy in cancer. *Allergy.* 2008;63(10):1255-1266.

26. Ishibashi S, Ohashi Y, Suzuki T, et al. Tumor-associated tissue eosinophilia in human esophageal squamous cell carcinoma. *Anticancer Res.* 2006;26(2b):1419-1424.

**Supplemental Table 1.** Diagnosis of esophageal, gastric and colorectal cancers*

| **Data source** | **Definition of cancer** | | |
| --- | --- | --- | --- |
| **UK Biobank** | **ICD-9 codes** | **ICD-10 codes** | **Self-reported cancer** |
| Esophageal cancer | 150, V10.03 | C15, Z85.01 | 1017 |
| Gastric cancer | 151, V10.04 | C16, Z85.028 | 1018 |
| Colorectal cancer | 153, 154.0, 154.1, V10.05, V10.06 | C18, C19, C20, Z85.038, Z85.048 | 1020, 1022, 1023 |
| **FinnGen consortium** | **ICD-8 codes** | **ICD-9 codes** | **ICD-10 codes** |
| Esophageal cancer | 150 | 150 | C15 |
| Gastric cancer | 151 | 151 | C16 |
| Colorectal cancer | 153,1540, 1541 | 153,154 | C18, C19, C20 |

*This information was not available for BioBank Japan.
